# Supplementary material for: Pregnancy pesticide exposure and child development in low- and middle-income countries: A prospective analysis of a birth cohort in rural Bangladesh and meta-analysis
Source: PLoS One. 2023 Jun 9;18(6):e0287089. doi: 10.1371/journal.pone.0287089 (PMC10256216; doi:10.1371/journal.pone.0287089)
Supplement: S1 Table — (DOCX) [file pone.0287089.s004.docx]

## **S1 Table** Search terms used in PubMed

**PubMed**

(“Child”[MH] OR “Child, Preschool”[MH] OR “child*”[tiab] OR “Adolescent”[MH] OR “adolescent”[tiab])

AND

(“Pesticides”[MH] OR “pesticide”[All fields] OR “Organophosphates”[MH] OR “organophosphate”[All fields] OR “organophosphorus”[All fields] OR "Organophosphorus Compounds"[MH] OR "Organophosphate Poisoning"[tiab] OR “aryldialkylphosphatase”[tiab] OR "Cholinesterase Inhibitors"[MH] OR "Cholinesterase Reactivators"[MH] OR "poly(thiophene-3-acetic acid" OR "neurotoxic esterase" OR "parathion hydrolase, Flavobacterium balustinum" OR “8- (4- (4- ((hydroxyimino) methyl) -1-pyridiniumyl) butyl) -3- ((3-hydroxy-2-phenylpropanoyl) oxy) -8-methyl-8-azoniabicyclo- (3.2.1) octane”[All fields] OR “isocarbophos” OR “1- ((4- (aminocarbonyl) pyridinio) trimethylene) -2- ((hydroxyimino) methyl) pyridinium” OR “duodote” OR “anilofos” OR “diethylphosphoryloxime” OR "tributylmethyl phosphonium chloride" OR “diclofluanid” OR "chloroacetol phosphate" OR “crufomate” OR “phosphorylphosphatase” OR "fenitrothion phosphatase" OR "phosphoramidic acid" OR “4- AND (4-nitrobenzyl) pyridine” OR “soman” OR “sarin” OR "Pralidoxime Compounds"[MH] OR “armin” OR “dichlorvos” OR “chlorfenvinphos” OR “armin” OR "Carbamyl Phosphate"[MH] OR “iphos” OR “mevinphos” OR “monocrotophos” OR “naled” OR “azinphosmethyl” OR “chlorpyrifos” OR “coumaphos” OR “cystaphos” OR “diazinon” OR “dimethoate” OR “disulfoton” OR “fenitrothion” OR “fenthion” OR “fonofos” OR “leptophos” OR “malathion” OR “parathion” OR "methyl parathion"[MH] OR "Phenylphosphonothioic Acid, 2-Ethyl 2-(4-Nitrophenyl) Ester"[MH] OR “phorate” OR “phosmet” OR “temefos” OR “paraoxon” OR “phosphamidon” OR “phosphines” OR “trichlorfon” OR “tetrachlorvinphos” OR “tetraisopropylpyrophosphamide” OR “dimethylphosphate” OR “dimethylthiophosphate” OR “dimethyldithiophosphate” OR “diethylphosphate” OR "dimethyl thiophosphate" OR "dimethyl dithiophosphate" OR "dimethyl phosphate" OR "dimethyl thiophosphate" OR "dimethyl dithiophosphate" OR "diethyl phosphate" OR "diethyl thiophosphate" OR "diethyl dithiophosphate" OR "Ethyl chlorpyriphos" OR "methyl chlorpyriphos" OR “PNP” OR “tcpy” OR “3,5,6-trichloro-2-pyridinol”)

AND

("Child Development"[MH] OR “child development”[tiab] OR “early child development”[tiab] OR “early childhood development”[tiab] OR “ECD”[tiab] OR "Child Behavior"[MH] OR “child behavior?r”[tiab] OR "Cognition"[MH] OR "Executive Function"[MH] OR “executive function*”[tiab] OR "Emotional Intelligence"[MH] OR "Emotions"[MH] OR “emotions”[tiab] OR “attachment”[tiab] OR “attention”[tiab] OR “behavio?r*”[tiab] OR “cognition”[tiab] OR “cognitive development”[tiab] OR “cognitive”[tiab] OR “cognitive skills”[tiab] OR “neurocognitive”[tiab] OR “communication”[tiab] OR “emotional”[tiab] OR “language”[tiab] OR “language skills” [tiab] OR “language development” [tiab] OR “receptive” [tiab] OR “expressive” [tiab] OR “speech development” [tiab] OR “communication skills” [tiab] OR "Motor Skills"[MH] OR “motor skills”[tiab] OR “fine motor”[tiab] OR “gross motor”[tiab] “prosocial”[tiab] OR “reading”[tiab] OR “social”[tiab] OR “sociali?ation”[tiab] OR “socio?emotion*”[tiab] OR “neurodevelopment”[tiab] OR “neurobehavio?r”[tiab] OR “neurobehvario?ral”[tiab] OR “Intelligence”[MH] OR “intelligence”[tiab] OR “IQ”[tiab] OR “psychomotor”[tiab] OR “sensorimotor”[tiab] OR “attention”[tiab] OR “inhibition”[tiab] OR “impulse control”[tiab] OR “working memory”[tiab] OR “problem solving”[tiab] OR “self-regulation”[tiab] OR “prosocial”[tiab] OR “psychosocial development”[tiab] OR “numeracy”[tiab] OR “math”[tiab] OR “literacy”[tiab] OR “school readiness”[tiab] “academic achievement”[tiab] OR “mental health”[tiab] OR “internali?ation”[tiab] OR “externali?ation”[tiab] OR “internali?ing symptoms”[tiab] OR “externali?ing symptoms”[tiab] OR “stress”[tiab] OR “anxiety”[tiab] OR “depression”[tiab] OR “mental disorders”[tiab] OR “anxiety disorders”[tiab] OR “eating disorders”[tiab] OR “neurocognitive disorders”[tiab] OR “neurodevelopmental disorders”[tiab])
