# Supplementary material for: Pregnancy pesticide exposure and child development in low- and middle-income countries: A prospective analysis of a birth cohort in rural Bangladesh and meta-analysis
Source: PLoS One. 2023 Jun 9;18(6):e0287089. doi: 10.1371/journal.pone.0287089 (PMC10256216; doi:10.1371/journal.pone.0287089)
Supplement: S2 Table — (DOCX) [file pone.0287089.s005.docx]

## **S2 Table** Comparison of enrolment characteristics of mother-child pairs with pesticide data included in the analytic sample and mother-child pairs without pesticide data excluded from the analysis, birth cohort in rural Bangladesh

|  | **Mother-child pairs with pesticide data (N=284)** | **Mother-child pairs without pesticide data (N=1,329)** | **p-value for difference between groups** |
| --- | --- | --- | --- |
|  | Mean ± SD or N (%) | Mean ± SD or N (%) | Mean ± SD or N (%) |
| *Maternal and household characteristics* |  |  |  |
| Age, years | 23.1±4.2 | 22.9±4.2 | 0.39 |
| Completed secondary school or higher | 149 (52.5) | 676 (50.9) | 0.62 |
| Monthly household income >4000 tk (~$43) | 186 (65.5) | 739 (57.0) | 0.01 |
| Husband engaged in agricultural work | 86 (30.3) | 400 (30.1) | 0.96 |
| *Maternal dietary intake at 28 weeks of gestation* |  |  |  |
| Total energy intake (kcal/day) | 3,173.4±734.5 | 2,818.8±1370.5 | <0.01 |
| Fruit intake (g/day) | 129.4±64.2 | 120.5±87.6 | 0.11 |
| Vegetable intake (g/day) | 161.3±124.7 | 137.2±131 | <0.01 |
