# Supplementary material for: Pregnancy pesticide exposure and child development in low- and middle-income countries: A prospective analysis of a birth cohort in rural Bangladesh and meta-analysis
Source: PLoS One. 2023 Jun 9;18(6):e0287089. doi: 10.1371/journal.pone.0287089 (PMC10256216; doi:10.1371/journal.pone.0287089)
Supplement: S3 Table — (DOCX) [file pone.0287089.s006.docx]

## **S3 Table** Comparison of characteristics at follow-up of mother-child pairs with pesticide data included in the analytic sample and mother-child pairs without pesticide data excluded from the analysis, among the sub-sample of children assessed on the Bayley Scales of Infant and Toddler Development, birth cohort in rural Bangladesh

|  | **Mother-child pairs with pesticide data (N=284)** | **Mother-child pairs without pesticide data (N=532)** | **p-value for difference between groups** |
| --- | --- | --- | --- |
|  | Mean ± SD or N (%) | Mean ± SD or N (%) | Mean ± SD or N (%) |
| *Maternal and household characteristics* |  |  |  |
| Age, years | 23.1±4.2 | 22.9±4.2 | 0.61 |
| Completed secondary school or higher | 149 (52.5) | 283 (53.2) | 0.84 |
| Monthly household income >4000 tk (~$43) | 186 (65.5) | 220 (41.7) | <0.01 |
| Husband engaged in agricultural work | 86 (30.3) | 174 (32.7) | 0.48 |
| *Maternal dietary intake at 28 weeks of gestation* |  |  |  |
| Total energy intake (kcal/day) | 3,173.4±734.5 | 3,344.0±986.0 | 0.01 |
| Fruit intake (g/day) | 129.4±64.2 | 140.4±93.7 | 0.08 |
| Vegetable intake (g/day) | 161.3±124.7 | 169±141.3 | 0.44 |
| *Child characteristics* |  |  |  |
| Female | 137 (48.2) | 260 (48.9) | 0.86 |
| Age at assessment, months | 26.5±1.9 | 29.0±3.0 | <0.01 |
| Cognitive composite score | 86.8±6.3 | 82.6±7.4 | <0.01 |
| Language composite score | 88.0±7.5 | 85.8±7.5 | <0.01 |
| Motor composite score | 88.2±6.5 | 83.3±7.9 | <0.01 |
