# Supplementary material for: Pregnancy pesticide exposure and child development in low- and middle-income countries: A prospective analysis of a birth cohort in rural Bangladesh and meta-analysis
Source: PLoS One. 2023 Jun 9;18(6):e0287089. doi: 10.1371/journal.pone.0287089 (PMC10256216; doi:10.1371/journal.pone.0287089)
Supplement: S4 Table — (DOCX) [file pone.0287089.s007.docx]

**S4** **Table** Associations between creatinine-adjusted prenatal pesticide biomarker concentrations (μg/g creatinine) and child development at 20-to-40-months of age, birth cohort in rural Bangladesh^1^

|  |  | **Cognitive** | | **Language** | | **Motor** | |
| --- | --- | --- | --- | --- | --- | --- | --- |
|  |  | Unadjusted  MD (95% CI) | Adjusted  MD (95% CI) | Unadjusted  MD (95% CI) | Adjusted  MD (95% CI) | Unadjusted  MD (95% CI) | Adjusted  MD (95% CI) |
| TCPY | Tercile 1 (n=97) 0.37 to 2.09 μg/g creatinine | Ref | Ref | Ref | Ref | Ref | Ref |
|  | Tercile 2 (n=93) 1.10 to 3.88 μg/g creatinine | -0.01 (-1.81, 1.78) | 0.12 (-1.67, 1.92) | 0.80 (-1.34, 2.93) | 0.53 (-1.55, 2.60) | -0.15 (-2.02, 1.71) | -0.02 (-1.54, 1.50) |
|  | Tercile 3 (n=94) 3.88 to 795.37 μg/g creatinine | -1.15 (-2.95, 0.64) | -1.08 (-2.92, 0.77) | -0.50 (-2.63, 1.62) | -0.87 (-3.00, 1.26) | 0.10 (-1.76, 1.96) | -0.32 (-1.89, 1.25) |
| 4-nitrophenol | Tercile 1 (n=95) 1.06 to 13.09 μg/g creatinine | Ref | Ref | Ref | Ref | Ref | Ref |
|  | Tercile 2 (n=94) 13.12 to 24.44 μg/g creatinine | 0.70 (-1.10, 2.51) | 0.15 (-1.67, 1.97) | 0.16 (-1.99, 2.30) | -0.22 (-2.32, 1.88) | 0.52 (-1.31, 2.35) | -0.13 (-1.66, 1.41) |
|  | Tercile 3 (n=95) 24.59 to 293 μg/g creatinine | 1.32 (-0.48, 3.11) | 0.63 (-1.23, 2.50) | -0.54 (-2.68, 1.60) | -0.77 (-2.93, 1.40) | **-2.46 (-4.29, -0.64)** | -0.98 (-2.56, 0.59) |
| IMPY | < LOD (n=239) | Ref | Ref | Ref | Ref | Ref | Ref |
|  | ≥ LOD (n=45) 0.14 to 7.62 μg/g creatinine | -1.06 (-3.07, 0.96) | -0.69 (-2.71, 1.33) | -1.72 (-4.1, 0.66) | -1.71 (-4.03, 0.62) | -0.99 (-3.07, 1.09) | -1.08 (-2.79, 0.63) |
| 3-PBA | < LOD (n=229) | Ref | Ref | Ref | Ref | Ref | Ref |
|  | ≥ LOD (n=55) 0.17 to 7.57 μg/g creatinine | 0.95 (-0.91, 2.81) | 0.91 (-0.95, 2.78) | 1.58 (-0.62, 3.78) | 1.88 (-0.26, 4.01) | -0.34 (-2.27, 1.58) | 0.55 (-1.02, 2.13) |

^1^ Estimates significant at 5% level in bold.

Adjusted models control for child age, child sex, maternal age, maternal education, maternal dietary intake, household income, and husband’s occupation. Abbreviations: TCPY, 3,5,6-trichloro-2-pyridino; IMPY, 2-isopropyl-4-methyl-6-hydroxypyrimidine; 3-PBA, 3-phenoxybenzoic acid; LOD, limit of detection; MD, mean difference; CI, confidence interval
