# Supplementary material for: Pregnancy pesticide exposure and child development in low- and middle-income countries: A prospective analysis of a birth cohort in rural Bangladesh and meta-analysis
Source: PLoS One. 2023 Jun 9;18(6):e0287089. doi: 10.1371/journal.pone.0287089 (PMC10256216; doi:10.1371/journal.pone.0287089)
Supplement: S5 Table — (DOCX) [file pone.0287089.s008.docx]

**S5** **Table** Heterogeneity of the adjusted associations between creatinine-adjusted prenatal pesticide biomarker concentrations (μg/g creatinine) and child development at 20-to-40-months of age by child sex, maternal education, household income, and husband’s occupation, birth cohort in rural Bangladesh^1^

|  | **TCPY** | | | **4-nitrophenol** | | | **IMPY** | | | **3-PBA** | | |
| --- | --- | --- | --- | --- | --- | --- | --- | --- | --- | --- | --- | --- |
|  | **Cognitive** | **Language** | **Motor** | **Cognitive** | **Language** | **Motor** | **Cognitive** | **Language** | **Motor** | **Cognitive** | **Language** | **Motor** |
|  | MD (95% CI) | MD (95% CI) | MD (95% CI) | MD (95% CI) | MD (95% CI) | MD (95% CI) | MD (95% CI) | MD (95% CI) | MD (95% CI) | MD (95% CI) | MD (95% CI) | MD (95% CI) |
| Child sex |  |  |  |  |  |  |  |  |  |  |  |  |
| Male | -0.02  (-0.03, -0.01) | **0.00**  **(-0.02, 0.01)** | -0.01  (-0.02, 0.00) | 0.01  (-0.03, 0.05) | -0.03  (-0.08, 0.02) | -0.02  (-0.06, 0.02) | 0.55  (-0.67, 1.77) | 0.22  (-1.21, 1.65) | **0.86**  **(-0.19, 1.90)** | 0.43  (-0.50, 1.35) | 0.31  (-0.79, 1.40) | **0.63**  **(-0.18, 1.43)** |
| Female | -1.59  (-3.07, -0.11) | **1.39**  **(-0.36, 3.15)** | -0.91  (-2.22, 0.39) | -1.03  (-3.00, 0.95) | 0.66  (-1.68, 3.00) | -1.27  (-3.00, 0.47) | -1.21  (-2.86, 0.44) | 0.93  (-1.00, 2.87) | **-0.68**  **(-2.09, 0.73)** | -1.23  (-2.84, 0.39) | 1.12  (-0.79, 3.03) | **-0.67**  **(-2.08, 0.73)** |
| p-value for interaction | 0.61 | **0.04** | 0.13 | 0.43 | 0.80 | 0.87 | 0.40 | 0.13 | **<0.01** | 0.47 | 0.40 | **0.02** |
| Maternal education |  |  |  |  |  |  |  |  |  |  |  |  |
| No formal schooling or primary school only | -0.02  (-0.10, 0.06) | -0.08  (-0.18, 0.02) | -0.07  (-0.14, 0.01) | 0.00  (-0.03, 0.03) | -0.02  (-0.05, 0.02) | **0.00**  **(-0.02, 0.03)** | 0.19  (-0.57, 0.95) | -1.08  (-1.97, -0.20) | -0.91  (-1.57, -0.25) | 0.17  (-0.44, 0.79) | -0.11  (-0.84, 0.62) | -0.24  (-0.78, 0.30) |
| Completed secondary school or higher | 1.38  (-0.17, 2.93) | 3.81  (1.96, 5.67) | 1.77  (0.40, 3.14) | 1.65  (-0.41, 3.71) | 4.84  (2.41, 7.26) | **3.60**  **(1.82, 5.38)** | 1.45  (-0.25, 3.15) | 3.31  (1.32, 5.31) | 1.24  (-0.23, 2.72) | 1.50  (-0.14, 3.15) | 4.14  (2.19, 6.09) | 1.85  (0.41, 3.29) |
| p-value for interaction | 0.94 | 0.14 | 0.12 | 0.62 | 0.48 | **0.02** | 0.69 | 0.11 | 0.14 | 0.90 | 0.83 | 0.59 |
| Household income |  |  |  |  |  |  |  |  |  |  |  |  |
| ≤4000 tk (~$43) | -0.21  (-0.56, 0.15) | -0.18  (-0.60, 0.25) | -0.04  (-0.35, 0.28) | 0.03  (-0.03, 0.08) | 0.02  (-0.04, 0.09) | -0.02  (-0.06, 0.03) | -0.09  (-2.27, 2.08) | -1.79  (-4.34, 0.76) | -0.25  (-2.14, 1.64) | 0.09  (-1.01, 1.18) | **1.25**  **(-0.03, 2.53)** | **0.89**  **(-0.06, 1.84)** |
| >4000 tk (~$43) | -0.36  (-2.38, 1.65) | -0.08  (-2.49, 2.34) | 0.67  (-1.12, 2.46) | 1.24  (-1.09, 3.57) | 2.06  (-0.68, 4.79) | 0.72  (-1.32, 2.77) | 0.17  (-1.98, 2.33) | -0.76  (-3.29, 1.76) | 0.45  (-1.42, 2.32) | 0.30  (-1.60, 2.20) | **1.48**  **(-0.75, 3.70)** | **1.44**  **(-0.21, 3.09)** |
| p-value for interaction | 0.30 | 0.43 | 0.86 | 0.22 | 0.11 | 0.99 | 0.84 | 0.39 | 0.66 | 0.89 | **0.02** | **0.01** |
| Husband's occupation |  |  |  |  |  |  |  |  |  |  |  |  |
| Not engaged in agriculture | -0.02  (-0.04, -0.01) | -0.01  (-0.02, 0.01) | -0.01  (-0.02, 0.00) | 0.00  (-0.03, 0.03) | -0.03  (-0.06, 0.01) | -0.01  (-0.04, 0.01) | 0.16  (-0.53, 0.85) | -0.75  (-1.56, 0.06) | -0.77  (-1.37, -0.17) | 0.08  (-0.54, 0.70) | -0.22  (-0.95, 0.51) | -0.34  (-0.88, 0.21) |
| Engaged in agriculture | 0.17  (-1.64, 1.97) | 0.75  (-1.41, 2.91) | -0.62  (-2.22, 0.98) | 0.25  (-2.13, 2.63) | -0.26  (-3.06, 2.54) | -0.16  (-2.24, 1.92) | 0.11  (-1.68, 1.90) | -0.32  (-2.43, 1.79) | -1.13  (-2.68, 0.43) | 0.18  (-1.57, 1.94) | 0.25  (-1.82, 2.32) | -0.83  (-2.37, 0.70) |
| p-value for interaction | 0.60 | 0.46 | 0.95 | 0.81 | 0.54 | 0.60 | 0.70 | 0.81 | 0.26 | 0.60 | 0.43 | 0.21 |

^1^ Estimates significant at 5% level in bold.

Adjusted models control for child age, child sex, maternal age, maternal education, maternal dietary intake, household income, and husband’s occupation. Abbreviations: TCPY, 3,5,6-trichloro-2-pyridino; IMPY, 2-isopropyl-4-methyl-6-hydroxypyrimidine; 3-PBA, 3-phenoxybenzoic acid; MD, mean difference; CI, confidence interval
